# Current Developments in Pt(IV) Prodrugs Conjugated with Bioactive Ligands

**DOI:** 10.1155/2018/8276139

**Published:** 2018-10-01

**Authors:** Xuejiao Li, Yahong Liu, Hongqi Tian

**Affiliations:** ^1^Tianjin Key Laboratory of Radiation Medicine and Molecular Nuclear Medicine, Institute of Radiation Medicine, Chinese Academy of Medical Sciences and Peking Union Medical College, Tianjin 300192, China; ^2^Tianjin Binjiang Pharma, Inc., Tianjin 300192, China

## Abstract

To overcome the side effects of and resistance to cisplatin, a variety of Pt(IV) prodrugs were designed and synthesized via different modifications including combination with lipid chains to increase hydrophobicity, conjugation with short peptide chains or nanoparticles to improve drug delivery, or addition of bioactive ligands to the axial positions of Pt(IV) complexes to exert dual-function effects. This review summarizes the recent progress in the development of Pt(IV) prodrugs conjugated with bioactive-targeting ligands, including histone deacetylase inhibitors, p53 agonists, alkylating agents, and nonsteroidal anti-inflammatory agents. Although Pt(IV) complexes that conjugated with bioactive ligands show satisfactory anticancer effects, none has been approved for clinical use. Therefore, we hope that this review will contribute to further study and development of Pt(IV) complexes conjugated with bioactive and other ligands.

## 1. Introduction

**Figure 1:**
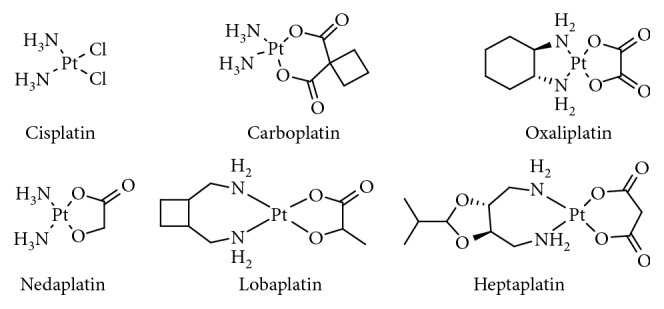
Pt(II) anticancer drugs approved for treatment.

Since the antitumor activity of cisplatin was serendipitously discovered by Rosenberg et al. in 1965 [1, 2], development of platinum anticancer agents has attracted the attention of numerous investigators. Although relatively few agents have been used as drugs clinically, the clinical value created is immeasurable. Currently, three platinum-based anticancer drugs, cisplatin, carboplatin, and oxaliplatin, have been approved by the U.S. Food and Drug Administration (FDA) and are used worldwide [3–5]. In addition, nedaplatin, heptaplatin, and lobaplatin have been approved for use in Japan, China, and South Korea, respectively [6]. The structures of these platinum drugs are shown in Figure 1.

According to statistics, platinum-based anticancer agents are clinically used to treat 50% of malignant cancers including testicular, ovarian, cervical, breast, and bladder cancers, as well as cancers of the head and neck, esophageal and lung cancers, mesothelioma, brain tumors, and neuroblastoma [6, 7]. When platinum-based drugs enter cells by passive or facilitated diffusion or active transport and are activated by aquation, their antineoplastic effects are linked to multiple mechanisms such as oxidative stress and mitochondrial DNA damage [8, 9]. However, their primary mechanism is attacking genomic DNA by covalently binding to the N7 position of guanine and adenine to form nuclear DNA adducts. This process inhibits transcription and replication, resulting in cell apoptosis.

Unfortunately, Pt(II)-based complexes, with their original structure-activity relationship summarized by Cleare and Hoeschele in 1973 [10], have numerous drawbacks including low bioavailability, severe side effects, poor stability, and inherent or acquired resistance [11]. Although multiple nonclassical Pt(II)-based complexes were designed and explored to overcome the shortcomings, such as *trans*-platinum, monofunctional, or polynuclear platinum complexes, no great success has been achieved yet.

To overcome the drawbacks of Pt(II)-based complexes, Pt(IV) complexes have been designed and synthesized as prodrugs. Pt(IV) complexes exhibit kinetic inertness and a low-spin *d*^6^ octahedral geometry, rendering them more stable for oral administration. Furthermore, modification of axial ligands can enhance pharmacological properties and reduce side effects and drug resistance [12]. In cells, Pt(IV) complexes are activated by reductive elimination to release active products; namely, Pt(II) drugs, which exert antitumor activity but in contrast to cisplatin, are not hydrolyzed or ligand-substituted [13]. Satraplatin represents the most successful Pt(IV) prodrug, which progressed to a phase III clinical trial of hormone-refractory prostate cancer (PC); however, the overall survival was not significantly improved. Ormaplatin (tetraplatin), iproplatin, and LA-12 have also progressed to clinical studies [14].

The current strategies for development of Pt(IV) complexes can be categorized into attachment of Pt(IV) to nanoparticles to increase bioavailability; synthesis of targeted Pt(IV) complexes to increase lipophilicity, facilitate cellular accumulation, and reduce side effects; and development of theranostic and photoactivatable prodrugs [7, 15, 16]. In this review, we focus on bifunctional targeting Pt(IV) prodrugs, paying particular attention to their molecular targets and antitumor effects. Although brief summaries of bifunctional Pt(IV) prodrugs have been provided in several articles [7, 12, 17], no review has provided an in-depth discussion of design details of targeted molecules and enhancement of their antitumor effects. Therefore, we summarized in detail Pt(IV) drug targeting and improved efficacy over the last 5 years to lay a theoretical foundation for the development of targeted Pt(IV) prodrugs conjugated with bioactive ligands.

## 2. Bifunctional Pt(IV) Prodrugs

The rational design of bifunctional Pt(IV) prodrugs relies on the reduction reaction of Pt(IV) to yield the corresponding active Pt(II) species and a biologically active ligand, improving the accumulation and activity of platinum drugs or helping them overcome drug resistance. Presently, the main Pt(IV) cores are cisplatin, carboplatin, and oxaliplatin, while bioactive ligands include histone deacetylase (HDAC) inhibitors, cyclooxygenase (COX) inhibitors, p53 inhibitors, and casein kinase 2 (CK2) inhibitors. Here, we summarize the current progress in the development of Pt(IV) prodrugs with various bioactive ligands.

### 2.1. Targeting HDAC

The nucleosome is composed of four core histones (H3, H4, H2A, and H2B) wrapped around with 147 base pairs of DNA [18]. It is the fundamental unit of chromatin, which regulates various cell processes such as DNA condensation, transcription, repair, and replication. Therefore, the status of histones is a key player in DNA transcription [19]. One of the most important histone modifications is acetylation of lysine residues, which is regulated by the equilibrium between two groups of enzymes, histone acetyltransferases (HATs), and HDACs [20]. HATs catalyze acetylation of histone lysine residues and neutralize the positive charge of histone tails, resulting in a more open chromatin state and making it more accessible to the transcriptional apparatus. In contrast, HDACs catalyze the removal of acetyl groups from lysine residues of histone tails, leading to chromatin condensation and transcriptional repression [21]. In the last decades, numerous HDAC inhibitors have shown good anticancer activities via different processes in various cell types and on multiple targets [22–25]. Based on their chemical structures, HDAC inhibitors include short-chain fatty acids, hydroxamic acids, synthetic benzamides, and cyclic peptides, which exhibit various bioactivities and specificities. Interestingly, it has been reported that DNA-damaging agents such as platinum drugs showed improved therapeutic profiles in combination with HDAC inhibitors since the latter could expose nuclear DNA to cytotoxic DNA-damaging agents, enhancing the efficacy of platinum drugs [26–28].

#### 2.1.1. Pt(IV) Conjugated with Valproic Acid (VPA)

**Figure 2:**
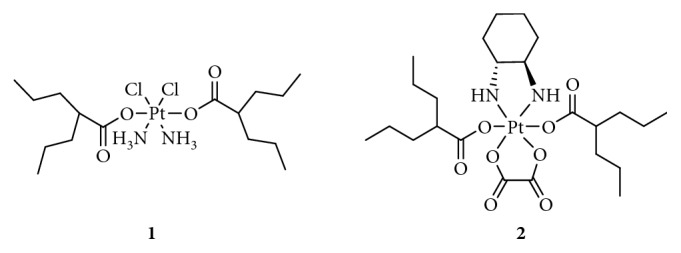
Structure of Pt(IV) prodrugs based on valproic acid (VPA).

Based on the above data, Yang et al. [29] have designed and synthesized a bifunctional Pt(IV) prodrug, VAAP (Figure 2(**1**)), based on oxidized cisplatin (diaminedichlorodihydroxyplatinum and ACHP) and an HDAC inhibitor, valproic acid (VPA), for cancer chemotherapy. The cytotoxicity of VAAP against A549, BCap-37, SKOV-3, and HepG2 cells was significantly higher than that of ACHP by 90-, 51-, 33-, and 73-fold, respectively. Furthermore, the cytotoxicity of VAAP was higher than that of cisplatin; thus, the half-maximal (50%) inhibitory concentration (IC_50_) value of VAAP was 12 times lower than that of cisplatin for the A549 cell line, while that of VPA was in the millimolar range, i.e., the same as reported in the literature [26, 30]. In addition, researchers have found that VAAP markedly induced cell death through apoptosis and cell-cycle arrest in the S phase. A mechanistic study has shown that although VPA facilitated the rapid entry of ACHP into cells, the cytosolic ACHP was rapidly removed. However, VAAP not only efficiently attached to the cell membrane but also improved the cytosolic platinum concentration, resulting in enhanced cytotoxicity. Remarkably, in comparison to the 50–100-fold improvement of the IC_50_ value, the cytosolic platinum concentration was only approximately twice that of ACHP after cells were treated with VAAP for 24 h. Studies have suggested that the high cytotoxicity of VAAP resulted from the combination of high cytosolic platinum concentration and synergy between the resulting Pt(II) and VPA. Unfortunately, the activity of HDAC was not tested in cells or a A549 tumor xenograft mouse model, although VAAP was confirmed to inhibit HDAC in the presence of ascorbic acid in a cell-free system. It is encouraging that VAAP significantly increased the antitumor activity, prolonged the blood circulation times, and decreased the nephrotoxicity in vivo compared with the effects of ACHP.

Alessio et al. [31] have synthesized VAAP again for the treatment of malignant pleural mesothelioma (MPM). Currently, the combination of cisplatin with the antifolate agent pemetrexed is considered a gold-standard frontline chemotherapy for MPM [32], while VAAP as a single drug displayed remarkable activity against a panel of cancer cells, especially MPM-derived cells in the study by Yang et al. [29]. The results of Alessio et al. [31] also indicated that the high lipophilicity of VAAP facilitated its passive diffusion, resulting in increased accumulation of platinum in the cells. Moreover, medium-chain fatty acid transport or caveolae-mediated endocytosis did not seem to be involved in this process. Interestingly, the authors demonstrated that there was no synergism, and the higher cytotoxicity of the Pt(IV) prodrug was solely due to the higher accumulation of Pt(II). The released intracellular VPA exhibited no specific effects on cellular biochemical processes since the concentration of VPA released by reduction was too low to exert any HDAC inhibitory activity, which contradicted the conclusions of Yang et al. [29]. In addition, Alessio and coworkers studied the biochemical behavior of Pt-VPA entrapped in liposomes and demonstrated much higher cellular accumulation of lipoPt-VPA; however, the antiproliferative effect increased only twofold, and thus, further studies of specific mechanisms are needed.

Because of the inconclusive reports on the role of HDAC inhibition in the mechanism of the toxicity of VAAP against tumor cells, Novohradsky et al. [33] reevaluated the mechanism of cytotoxicity of Pt(IV)-VPA derivatives, focusing on the previously published contradictory conclusions. These authors confirmed that the Pt(IV) derivatives of cisplatin conjugated with VPA accumulated in and killed cancer cells with a greater efficacy than that of cisplatin owing to the considerably higher binding of VPA-containing platinum to DNA. This superior effect was mainly attributed to increased hydrophobicity. Most importantly, cellularly, the Pt(IV) complexes remarkably inhibited HDAC activity, reduced the expression of HDAC enzymes, and induced distinct rates of histone H3 hyperacetylation in cisplatin-sensitive (A2780) and cisplatin-resistant (A2780cisR) ovarian cancer cells, resulting in chromatin decondensation. Moreover, the Pt(IV) derivatives of cisplatin conjugated with VPA reduced the level of glutathione (GSH) in tumor cells with more pronounced in A2780cisR than in A2780 cells. In summary, the results indicated that the enhanced antitumor effect resulted from both improved cellular accumulation of platinum and VPA, released by intracellular reduction of Pt(IV) prodrugs, and multiple bioactivities of VPA.

In addition, Novohradsky's team synthesized a new bifunctional Pt(IV) complex (Figure 2(**2**)) based on oxaliplatin [34] to overcome the limitation of the clinical use of cisplatin. Although the cytotoxicity of the Pt(IV) complex was lower than that of oxaliplatin, the results showed that free VPA released from the Pt(IV) complex reduced the activity of HDACs and upregulated the expression of the histone H3 protein complexed with DNA, leading to an enhanced cytotoxicity to tumor cells, which was attributed to the synergism between Pt(II) and VAP, released by reduction of the complex in tumor cells.

#### 2.1.2. Pt(IV) Complexes Conjugated with 4-Phenylbutyric Acid (PhB)

**Figure 3:**
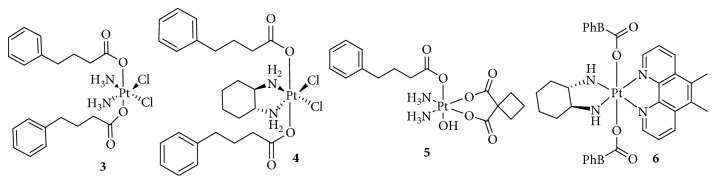
Structure of Pt(IV) prodrugs based on 4-phenylbutyric acid (PhB).

Using another HDAC inhibitor, 4-phenylbutyric acid (PhB), Raveendran et al. [35] synthesized a new class of Pt(IV) derivatives based on the cores of cisplatin and oxaliplatin (Figure 3(**3** and **4**)) and compared their bioactivities to those of Pt(IV) derivatives conjugated with VPA. The researchers found that the Pt(IV) derivatives of cisplatin were more potent than those of oxaliplatin, which was consistent with the data of previous reports. Pt(IV) derivatives of cisplatin with a *bis*-PhB derivative (VI) were approximately 5- and 100-fold more potent than a *bis*-VPA analog and cisplatin, respectively, suggesting that PhB was more effective than VPA in promoting cytotoxicity. Furthermore, the HDAC inhibitory activity of the *bis*-PhB analog in cancer cells was 60–70% at concentrations lower than the IC_50_ of PhB, indicating a synergism between platinum and PhB. The enhanced effect was associated with the rates of cellular accumulation and Pt-DNA binding, not with the hydrophobicity, as concluded based on logP values.

Further mechanistic studies have revealed that Pt(IV) derivatives conjugated with PhB induced tumor cell apoptosis through the mitochondrial pathway. This was associated with the activation of caspase-3, -9, and -7 (involved in the intrinsic apoptosis pathway) and the reduction of the mitochondrial membrane potential, which was p53-independent, unlike the mechanism of action of cisplatin. Therefore, the “dual-action” complexes are, in fact, “multiaction” complexes triggering several different pathways, which exert antitumor effects together. Recently, Almotairy et al. [36] prepared Pt(IV) complexes with PBA based on the carboplatin scaffold (Figure 3(**5**)); however, the increase in antitumor activity was lower than that induced by cisplatin analogs. In addition, Harper et al. [37] designed Pt(IV) derivatives of [Pt(1*S*,2*S*-diaminocyclohexane) (5,6-dimethyl-1,10-phenanthroline)] (Pt56MeSS, Figure 3(**6**)), with two PhBs, which displayed a relatively minor association between the axial ligands and the potency of the compounds, while the average IC_50_ values were better than that of cisplatin.

#### 2.1.3. Pt(IV) Complex Conjugated with Suberoyl-*bis*-Hydroxamic Acid (SubH)

**Figure 4:**
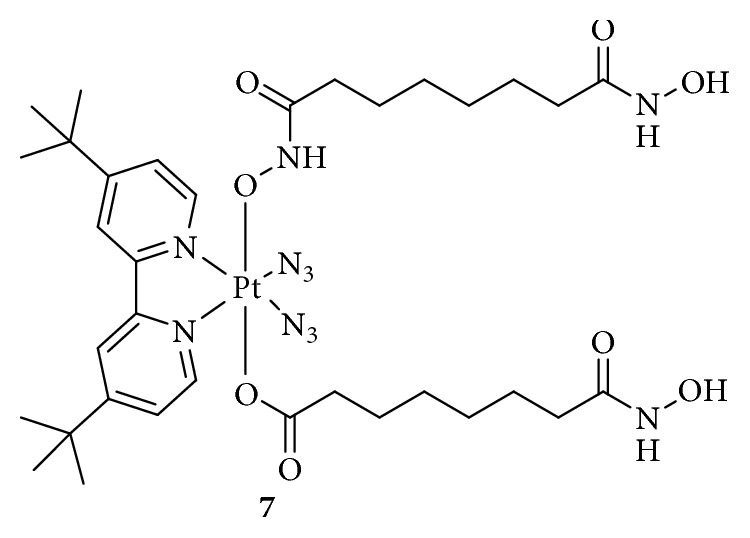
Structure of Pt(IV) prodrugs based on suberoyl-*bis*-hydroxamic acid (SubH).

Suberoyl-*bis*-hydroxamic acid (SubH) shows HDAC inhibitory activity at submicromolar concentrations and antitumor effects at micromolar concentrations. Furthermore, following its conjugation with Pt(II) complexes, SubH displayed potent synergistic interactions [38–40]. Interestingly, Kasparkova et al. [41] added two SubHs to a nontoxic Pt(IV)-azido complex to form a photoactivatable Pt(IV) complex (Figure 4(**7**)), which exhibited a higher efficacy in photodynamic anticancer chemotherapy. Following irradiation with ultraviolet A (UVA), the cytotoxicity of Pt(IV) significantly increased by 6–11-fold than that of cisplatin, which was not affected by light.

Under UVA or visible light, the Pt(IV) complex was activated, and the released Pt(II) was bound to DNA, while the released SubH inhibited HDAC activity, leading to improved H3 acetylation. More importantly, the activated SubH-conjugated Pt(IV) complex inhibited the prolongation of RNA transcription by blocking RNA polymerase II and destroying the global structure of DNA, which was not observed in the dark. This strategy based on the light-induced simultaneous HDAC inhibition and DNA damage by platinum drugs provided a new method for the development of “dual-action” Pt(IV) prodrugs.

### 2.2. Targeting p53

**Figure 5:**
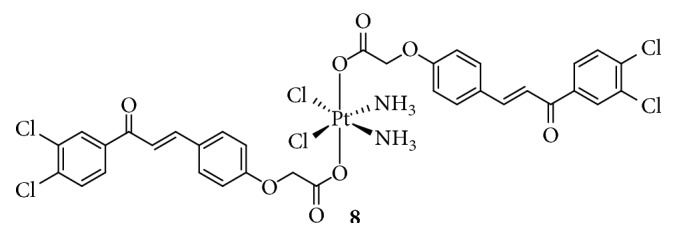
Structure of Pt(IV) prodrugs based on chalcone.

In 1989, tumor protein p53 was first reported by Finlay et al. [42] as an inhibitor of oncogene-mediated transformation, associated with cell-cycle arrest or apoptotic activities. Many studies have shown that p53-regulated cell cycle arrest or apoptosis is important for the inhibition of tumor development, and p53 is involved in multiple cellular processes such as oncogene activation, DNA damage, DNA repair, metabolism, and antioxidant responses [43]. Mutations in p53 contribute to tumor formation or drug resistance. In fact, p53 was named the “guardian angel” of genome integrity [44]. However, the human murine double minute 2 (MDM2) oncoprotein mediates and inhibits p53 function through direct interactions [45]. Therefore, a small molecule inhibitor designed to block the p53-MDM2 interaction might be effective in treating human cancers retaining wild-type p53. Chalcone, a well-known p53-MDM2 inhibitor, binds to the p53 pocket on MDM2 to inhibit the p53-MDM2 interaction at a micromolar range concentration [46]. Ma and colleagues [47] designed a novel, dual-targeting Pt(IV) anticancer prodrug, chalcoplatin, based on cisplatin and chalcone, which not only formed Pt-DNA adducts but also activated the p53 pathway (Figure 5(**8**)). The results showed that, compared to cisplatin, chalcoplatin exhibited a markedly higher cytotoxicity (10-fold) for wild-type p53 cells but not for p53 null cells. Although the cellular accumulation increased, the Pt-DNA binding rate was not improved. Moreover, chalcoplatin mainly induced cell cycle arrest in the S and G2/M phases, whereas cisplatin and chalcone caused cell cycle arrest in the S and G2/M phases, respectively. These results indicated that the chalcone moiety in chalcoplatin played a pivotal role in killing cancer cells, possibly by activating p53. In fact, chalcoplatin more effectively promoted the expression of p53 than cisplatin. Unfortunately, although chalcoplatin showed distinct effects on cisplatin-resistant A549 cells (IC_50_, 50.5, and 1.6 *μ*M for cisplatin and chalcoplatin, respectively), the specific mechanism was not studied.

### 2.3. Targeting DNA

**Figure 6:**
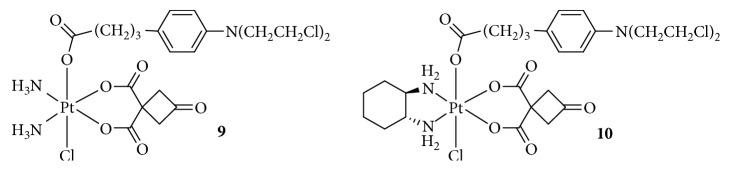
Structure of Pt(IV) prodrugs based on Chl.

DNA-damaging agents play a key role in anticancer chemotherapeutics and are widely used clinically [48]. In addition to platinum drugs, DNA-alkylating agents are essential components and include nitrogen mustards that represent the earliest and most widely studied DNA cross-linking agents such as cyclophosphamide, chlorambucil (Chl), and melphalan. Chl has been approved by the FDA and is primarily used in the treatment of chronic lymphocytic leukemia and other cancers, including non-Hodgkin lymphoma, certain types of trophoblastic neoplasms, and ovarian carcinoma [49–51]. Chl, which possesses an *N*,*N*-*bis*(2-chloroethyl)amine functional group, has reactive electrophiles and covalently binds to DNA at the N3 or N7 position of adenine or N7 position of guanine to inhibit DNA replication and transcription [52]. However, serious drug resistance and toxicities hindered its clinical application, similar to Pt(II) complexes. Based on this, Chen et al. [53, 54] synthesized a series of Pt(IV) derivatives by conjugating a Chl unit with cisplatin, oxaliplatin, and carboplatin analogs (Figure 6(**9** and **10**)). These Pt(IV) complexes not only exhibited significant antitumor activities but also overcame the drug resistance of cisplatin-resistant cancer cells. One of the most critical factors was that the free Chl released from the Pt(IV) complexes synergistically increased DNA damage induced by the released Pt(II) complexes. Moreover, Chl blocked the double-strand break repair by suppressing the double-strand break repair MRE11-RAD50-NBS1 (MRN) protein complex, decreasing CK2 phosphorylation, and reducing the expression of proline-rich acidic protein 1 (PRAP1). These effects increased the phosphorylation of histone H2A histone family, member X (H2AX), which is closely related to drug resistance in tumor cells. More importantly, some complexes exhibited a longer blood retention time and nearly no toxicities in vivo, suggesting their potential as platinum-based anticancer drug candidates.

**Figure 7:**
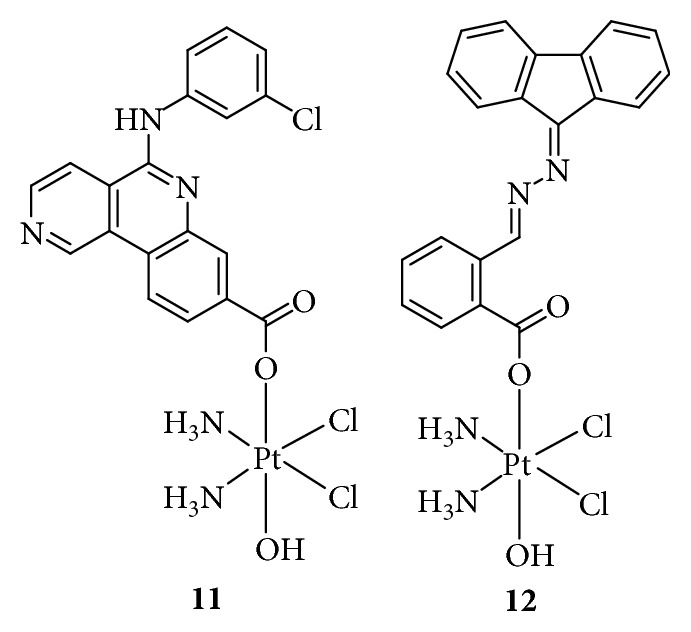
Structure of Pt(IV) prodrugs based on casein kinase 2 (CK2) and nucleotide excision repair (NER) inhibitors.

The DNA damage response (DDR) network plays a key role in the maintenance of genomic integrity and regulation of the efficacy of anticancer drugs. The DDR network mainly consists of DNA damage recognition and repair pathways, which involve multiple signaling molecules including MRN, ataxia telangiectasia mutated (ATM), ATM and Rad3-related protein (ATR), checkpoint kinase 1 (CHK1), X-ray repair cross-complementing protein 1 (XRCC1), mediator of DNA damage checkpoint protein 1 (MDC1), CK2, *γ*H2AX, WEE1 G2 checkpoint kinase, XPA DNA repair protein, excision repair 1 protein (ERCC1), and ERCC1-ERCC4 complex. These molecules are associated with survival and resistance and therefore, are attractive targets for cancer therapy. For example, Chen et al. [55] used the CK2 inhibitor CX-4945 as an axial ligand to design a Pt(IV) prodrug (Figure 7(**11**)), Cx-platin, which disrupted DDR through the suppression of CK2-phosphorylated MDC1, thereby combining the forkhead-associated domain of aprataxin with DNA double-strand breaks and finally inducing apoptosis in a panel of cancer cells, similar to Chl, in response to DNA damage. Wang et al. [56] also synthesized a Pt(IV) anticancer prodrug targeting nucleotide excision repair (NER) by adding a NER inhibitor, (*E*)-2-([{8a,9a-dihydro-9*H*-fluoren-9-ylidene}hydrazono]methyl)benzoic acid (Figure 7(**12**)), to axial positions of oxidized cisplatin to overcome the cisplatin resistance. The NER inhibitor-Pt(IV) complex showed a 22- and 3.54-fold higher cytotoxicity against A2780cisR and A549cisR cells, respectively, than against A2780 and A549 cells, by inducing high levels of Pt-GG intrastrand crosslinks. The model of biofunctional Pt(IV) prodrugs inhibiting DDR provides a strategy to conquer the resistance to cisplatin.

### 2.4. Targeting Hypoxia-Inducible Factor (HIF)

**Figure 8:**
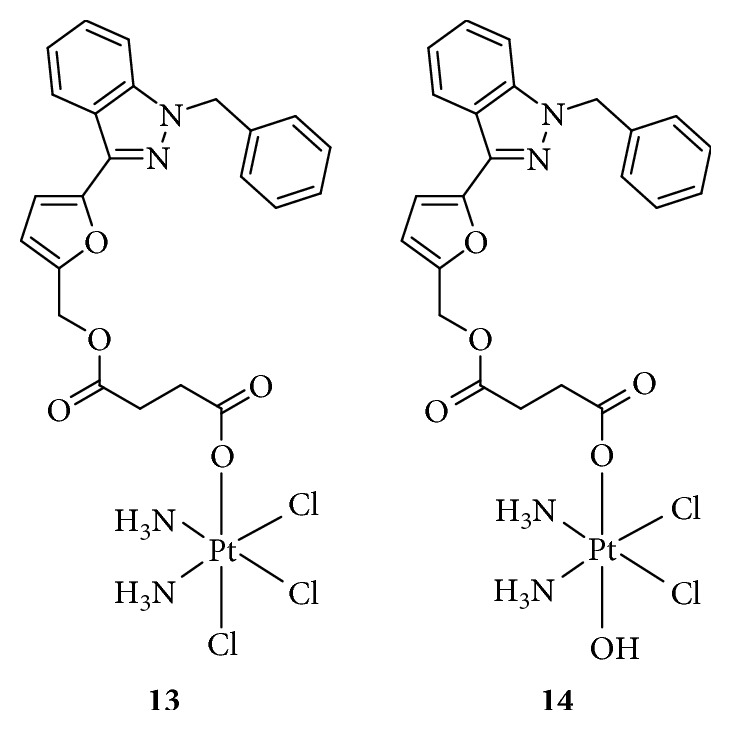
Structures of Pt(IV) prodrugs YCC-1 and YCC-2.

Hypoxia plays a critical role in the development of cancer and is caused by an inadequate supply of oxygen (median concentration, 0–4%) [57]. Hypoxia is a hallmark in many solid tumors. Approximately 70% of cancer cells respond to hypoxia by increasing the expression of the hypoxia-inducible factor (HIF)-1, which can translocate to the nucleus and activate the relative transcription of genes involved in tumorigenesis [58]. Unlike a normoxic microenvironment, the hypoxic tumor microenvironment can weaken the antitumor effects of Pt(II) drugs. Xu et al. [59] developed two novel Pt(IV) anticancer prodrugs, YCC-1 (Figure 8(**13**)) and YCC-2 (Figure 8(**4**)), which not only enhanced the platination of DNA but also inhibited the expression of HIF-1*α* via release of HIF-1*α* inhibitors from YCC-1 and, especially, from YCC-2 under hypoxia but not normoxia, thus significantly enhancing the sensitivity of tumor cells to cisplatin. The unique difference between the structures of YCC-1 and YCC-2 was that the axial chloride group of YCC-1 was substituted with a hydroxyl group in YCC-2, suggesting that axial ligands such as a chloride or hydroxyl group also affect the bioactivity of Pt(IV) complexes. In vivo, YCC-2 effectively inhibited HCT-116 tumor growth, without much toxicity, based on the slight weight change of mice. This study provided a new approach to increase the effect of chemotherapeutic drugs.

### 2.5. Targeting COX

**Figure 9:**
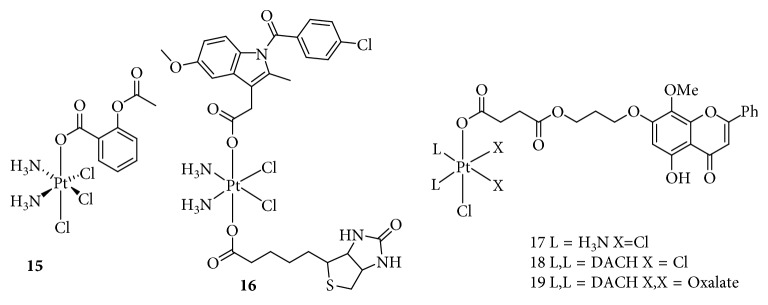
Structures of Pt(IV) prodrugs based on aspirin, biotin, and wogonin derivatives.

Chronic inflammation occurs in approximately 20% of human cancers and plays an important role in tumor growth and metastatic progression [60]. Clinical data have suggested that tumor progression is often accompanied by chronic inflammation related to increased COX expression, including COX-1 and COX-2, which are the crucial enzymes in prostaglandin biosynthesis [61]. Recently, the combination of anti-inflammatory and chemotherapeutic activities for the treatment of cancer has attracted increasing attention [62]. Pathak et al. [63] used the nonsteroidal anti-inflammatory drug (NSAID) aspirin and cisplatin to fabricate a Pt(IV) prodrug, platin-A, for the treatment of PC (Figure 9(**15**)). Platin-A showed cytotoxicity similar to that of cisplatin with a slightly higher IC_50_ value than that of an equimolar mixture of cisplatin and aspirin. However, platin-A markedly reduced the expression of COX-2 and the levels of tumor necrosis factor (TNF)-*α* and interleukin (IL)-6, which were increased in lipopolysaccharide- (LPS-) activated macrophages. Moreover, platin-A promoted the secretion of the anti-inflammatory cytokine IL-10 in LPS-activated macrophages, which was not observed in LPS-activated macrophages treated with cisplatin, aspirin, or an equimolar mixture of both. These results indicated that platin-A exhibited unique anti-inflammatory and antitumor effects. Cheng et al. [64] also reported a Pt(IV) prodrug, which was a fusion of aspirin and cisplatin, based on antineoplastic effects of aspirin. Compared to cisplatin, this Pt(IV) complex increased the in vitro cytotoxicity by approximately 10-fold and inhibited the tumor growth in vivo with a low systemic toxicity. Another Pt(IV) complex conjugated with an NSAID was reported by researchers from Gou's laboratory [65], who used cisplatin, biotin, and indomethacin to synthesize a biotin-Pt(IV)-indomethacin hybrid for cancer therapy (Figure 9(**16**)). This complex effectively targeted cancer cells owing to the presence of the biotin moiety, which was recognized by the biotin receptor overexpressed on the cell surface. In cells, the Pt(IV) complex was reduced to a DNA-binding Pt(II) complex, while the released indomethacin inhibited COX activity and modulated the cellular response to the platinum drug. In addition, the biotin-Pt(IV)-indomethacin complex decreased tumor invasiveness and destroyed capillary-like tube formation [66].

In addition, Gou's group [67, 68] also synthesized a series of novel Pt(IV) complexes with wogonin or a wogonin derivative conjugated at an axial position (Figure 9(**17**–**19**)). Wogonin, isolated from the root of the medicinal herb *Scutellaria baicalensis* Georgi, displays anti-inflammatory, antioxidant, and anticancer activities both in vitro and in vivo. The Pt(IV) complexes obtained inhibited the activity of COX-2 by blocking the CK2-mediated nuclear factor- (NF-) *κ*B pathway, which promotes tumor survival and invasion. The Pt(IV) complexes conjugated with the wogonin derivative were multitargeting anticancer agents, owing to the diverse biological activities of wogonin.

### 2.6. Targeting Mitochondria

**Figure 10:**
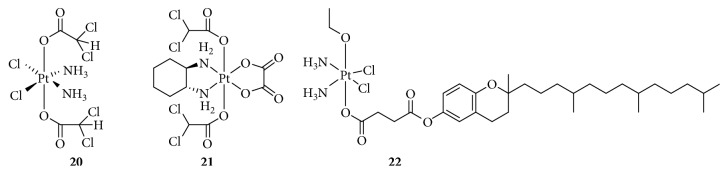
Structure of Pt(IV) prodrugs based on dichloroacetate (DCA) and vitamin E analog.

The mitochondria occupy 15–50% of the cytoplasmic volume in most cells and play key roles in cellular energy-generating processes and are considered main regulators of cell survival. Mitochondrial oxidative phosphorylation provides energy needs for normal cells, whereas cancer cells mainly rely on aerobic glycolysis (the Warburg effect) [69]. This unique glucose metabolic pathway of cancer suggests that the mitochondria could be a prime target for cancer therapy. Dhar and Lippard [70] presented a Pt(IV) prodrug, mitaplatin (Figure 10(**20**)), a potent fusion of cisplatin and the orphan drug dichloroacetate (DCA), and explored its bioactivity. DCA, as an anticancer agent without any deleterious effect on normal cells, reversed the Warburg effect [71, 72] by inhibiting pyruvate dehydrogenase kinase (PDK), which is required for this process, which opened the mitochondrial transition pores. This effect released proapoptotic mediators such as cytochrome c and induced the translocation of apoptosis-inducing factor (AIF) to induce apoptosis. Mitaplatin displayed a dual killing activity against cancer cells, which included DNA damage by cisplatin and selective mitochondrial damage by DCA in cancer cells. Mitaplatin not only showed high cytotoxicity against tested cancer cells but also decreased the mitochondrial membrane potential gradient, thus, inducing apoptosis by promoting the release and translocation of cytochrome c and AIF.

However, Zajac and colleagues [73] have demonstrated that mitaplatin, with a half-life of less than 1 h, is not stable in the cell culture medium for the entire period required for in vitro cytotoxicity studies (24, 48, or 72 h). Thus, the observed biological effects were due to the action of a mixture of mitaplatin and its hydrolysis products. Therefore, researchers designed a new Pt(IV) complex based on DCA and oxaliplatin. The antitumor model was similar to that of the conjugation of cisplatin and DCA, while 5-fluorouracil in combination with DCA-containing Pt(IV) derivatives of oxaliplatin enhanced the antitumor effect (Figure 10(**21**)).

In addition, Suntharalingam et al. [74] have reported the synthesis of Pt(IV) complexes conjugated with a vitamin E analog, *α*-tocopherol succinate (Figure 10(**22**)), which disrupted mitochondrial function by inhibiting B-cell lymphoma- (BCL-) extra-large (BCL-xL) and BCL-2 proteins. These two proteins are important components of the mitochondrial apoptosis pathway [75]. The results showed that the cytotoxicity was improved 7–220-fold, and the expression of BCL-xL and BCL-2 was decreased by the Pt(IV) prodrugs, suggesting that the dual targeting strategy could be a valuable route for increasing the anticancer efficacy.

**Figure 11:**
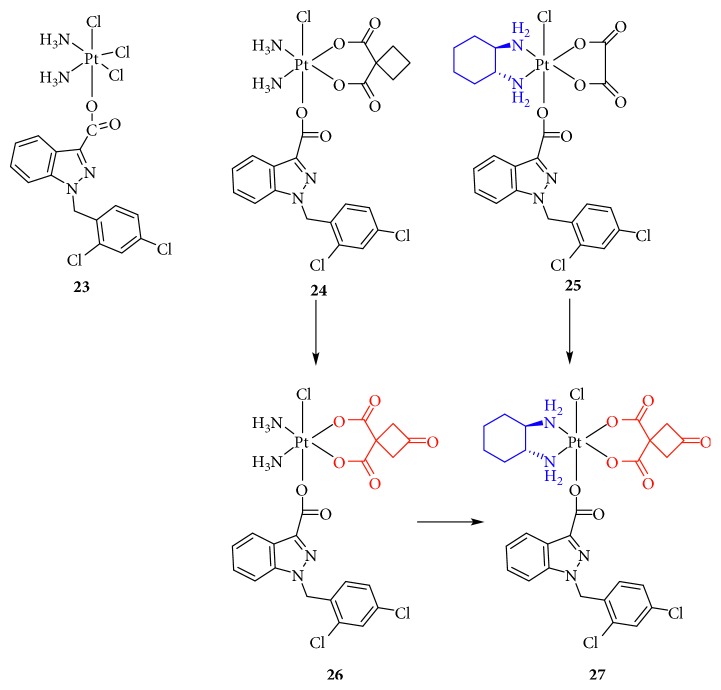
Structure of Pt(IV) prodrugs based on lonidamine (LND).

Recently, Chen and colleagues [76] developed five new Pt(IV) prodrugs based on cisplatin, carboplatin, oxaliplatin, and their derivatives conjugated with lonidamine (LND) as an axial ligand, which is known to interfere with cancer metabolism by targeting the glycolytic pathway (Figure 11(**23**–**27**)). The results showed that all these complexes enhanced antitumor effects against different tumors, especially the Pt(IV) prodrug based on cisplatin, which exhibited significantly improved anticancer activities against LNCaP that was nearly twice as high as that of cisplatin and showed considerably more potential than the physical mixture of cisplatin and LND. Further research showed that this complex was easily reduced by ascorbic acid to release LND at room temperature and triggered cancer cell death via an apoptotic pathway, but with a low cellular uptake of platinum, suggesting that the induction of apoptosis in LNCaP cells was closely associated with mitochondrial function disruption and ROS accumulation, which was associated with LND release.

In summary, these results provide evidence to support the design strategy of conjugating platinum complexes with mitochondria-targeting inhibitors to improve their anticancer effect.

### 2.7. Targeting Microtubules

**Figure 12:**
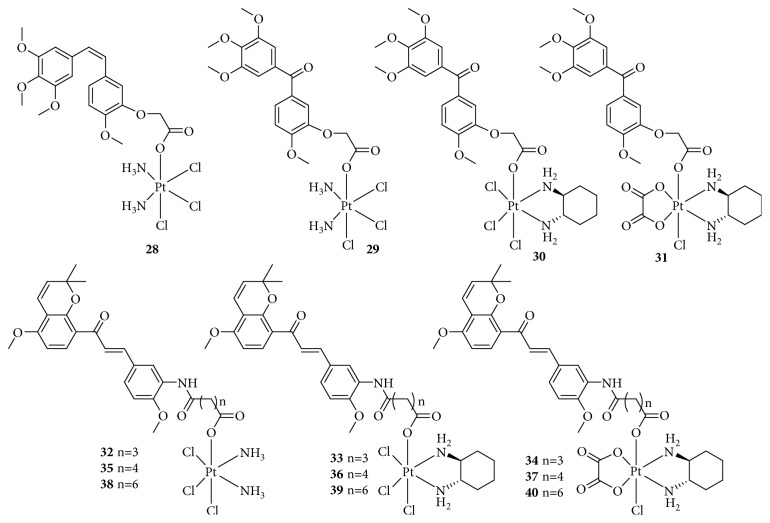
Structure of Pt(IV) prodrugs based on combretastatin A-4, phenstatin analogs, and millepachine analogs.

Microtubules, composed of *α*,*β*-tubulin heterodimers and well known as a major part of the cytoskeleton, play an important role in many critical cellular processes such as cell signaling, mitotic spindle assembly during cell division, intracellular transport, cell proliferation, and migration [77, 78]. Inhibition of the assembly of tubulin into microtubules or depolymerization of microtubules leads to apoptosis via arrest of cell division, rendering microtubules an attractive molecular target for antitumor agents [79]. It has been reported that combining cytotoxic, DNA-damaging platinum-based drugs with tubulin inhibitors increases antiproliferative activities. Wang and colleagues [80, 81] designed a series of Pt(IV) complexes comprising the tubulin inhibitor combretastatin A-4 or phenstatin analogs as part of dual-targeting Pt(IV) prodrugs derived from cisplatin, oxaliplatin, and dichloro(1,2-diaminocyclohexane)platinum(II) (Figure 12(**28**–**31**)). These Pt(IV) complexes, conjugated with tubulin inhibitors, showed a good cytotoxicity for cancer cells, including those with cisplatin resistance. Moreover, the complexes effectively accumulated in cells, strongly inhibited tubulin polymerization, blocked the cell cycle at the G2/M phase, and markedly induced cell apoptosis through the mitochondrial pathway. In NCI-H460 and HepG2 xenograft mouse models, although these complexes also inhibited the tumor growth *in vivo*, the activity was slightly less than that of cisplatin. Therefore, they also designed and synthesized a series of new Pt(IV) prodrugs with millepachine analogs (Figure 12(**32**–**40**)), which have been found to exhibit potent inhibitory effects on tubulin polymerization by binding to the colchicine site of tubulin [82]. These compounds are expected not only to carry the DNA damaging platinum-based agent warhead into the tumor cells but also have a millepachine derivative that inhibits tubulin polymerization. Furthermore, *in vivo* tests showed that this kind of compound effectively inhibited tumor growth without obvious body weight loss in an SK-OV-3 xenograft model in contrast to the reference drugs Pt(II) agents and millepachine. In general, the combination of millepachine analogs Pt(II) agents is a useful strategy to inhibit tumor growth with low toxicity.

### 2.8. Targeting Vascular Processes

**Figure 13:**
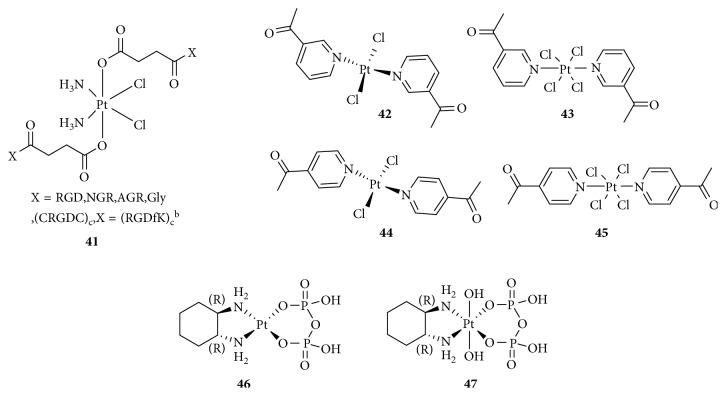
Structure of Pt(IV) prodrugs based on Arg-Gly-Asp (RGD), Asn-Gly-Arg (NGR), and acetylpyridine.

Angiogenesis promotes tumor growth and is involved in cancer cell development, including survival and metastasis [83]. This process requires the formation of new vessels [84]. Cancer cells promote angiogenesis early in tumorigenesis, which is driven by an oncogene to express proangiogenic proteins such as the vascular endothelial growth factor (VEGF), basic fibroblast growth factor (bFGF), IL-8, placenta-like growth factor (PLGF), and transforming growth factor- (TGF-) *β*. [85]. Targeting vascular endothelial cells and blocking tumor angiogenesis is a relatively new approach to cancer therapy. Mukhopadhyay and colleagues [86] used peptides containing Arg-Gly-Asp (RGD) and Asn-Gly-Arg (NGR, Figure 13(**41**)), which were added to an axial position to form a series of Pt(IV)-peptide complexes for targeting the angiogenic tumor vasculature. RGD and NGR can be recognized by integrins *α*_v_*β*_3_ and *α*_v_*β*_5_ and the membrane-spanning surface protein aminopeptidase *N*, respectively, which are highly expressed in tumor-induced angiogenesis [87]. The RGD- and NGR-tethered Pt(IV) complexes showed better inhibition of cellular proliferation than nonspecific Pt(IV)-peptide analogs, suggesting selective transport of platinum anticancer agents into tumor endothelial cells. Lana [88] also synthesized four antivascular *trans*-Pt(II)/(IV) complexes with acetylpyridine as a ligand (Figure 13(**42**–**45**)). These complexes exhibited antiangiogenesis effects, whereas the Pt(II) complexes were more effective than the Pt(IV) complexes.

It is worth mentioning that Yang et al. [89] reported two phosphaplatins, a Pt(IV) drug without a bioactive ligand (1R,2R-diaminocyclohexane)(dihydropyrophosphato)(transdihydroxo) Pt(IV) (RRD4, Figure 13(**47**)) and a Pt(II) drug (RRD2, Figure 13(**46**)), targeting VEGF receptor (VEGFR-2 instead of DNA). These phosphaplatins markedly inhibited both cell migration and tube formation in vitro and tumor vascularization in vivo, and moreover, the activities of RRD2 were better than that of RRD4, whereas cisplatin and carboplatin did not. The researchers proposed that the mechanism of phosphaplatin's antiangiogenic activity was that the sulfur-containing protein side chains of VEGFR-2 may form metal-ligand complexes with phosphaplatins through displacement of the Pt pyrophosphate ligand. Unfortunately, phosphaplatins are not susceptible to aquation at pH 7.4, and greater solution stability may play a major role in determining their ultimate biological targets.

### 2.9. Targeting GSH-S-Transferase

**Figure 14:**
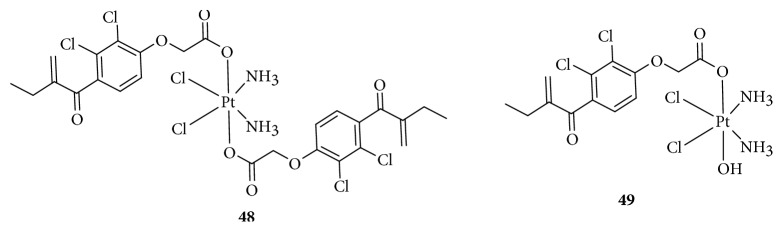
Structure of Pt(IV) prodrugs based on ethacrynic acid (EA).

Drug resistance is one of the most serious factors limiting clinical applications of cisplatin, which is involved in multifactorial processes. GSH-S-transferase (GST) enzymes have been demonstrated to be the most important factors of drug resistance. They are well known to catalyze the conjugation of GSH to cisplatin in cells to detoxify cisplatin, resulting in drug resistance [88, 90]. Studies have shown that GST enzymes are overexpressed in various cisplatin-resistant cells, especially pi class GST (GSTP1-1), an important enzyme in the mercapturic acid detoxification pathway. The inhibition of GST activity could reverse drug resistance. It has been reported that a GST inhibitor (ethacrynic acid, EA) combined with alkylating agents (Chl and thiotepa) for the treatment of tumor showed some success [91]. Therefore, researchers used Pt(IV) compounds conjugated to EA to overcome the GST-mediated drug resistance (Figure 14(**48**)). The Pt(IV)-EA complexes not only strongly inhibited the GST activity in cell-free and cell systems (∼10-fold of EA alone) but also rapidly kill cancer cells. To understand the molecular mechanism, Parker and colleagues [92] investigated the drug-protein interactions between Pt(IV)-EA and GST P1-1, supported by molecular modeling analysis using quantum mechanical/molecular mechanical methods. The results showed that EA-CPT preferentially docks at the dimer interface at GST P1-1 and subsequent interaction with the enzyme resulted in docking of the ethacrynate ligands at both active sites (in the H-sites), with the Pt moiety remaining bound at the dimer interface. The activation of the inhibitor by its target enzyme and covalent binding accounts for the strong and irreversible inhibition of enzymatic activity by the platinum complex. Interestingly, Zanellato et al. [93] found that Pt(IV and II)-ethacrynic acid conjugates showed poorer performance than the reference drug cisplatin alone or in combination with EA did in the treatment of MPM, and cellular GST activity remained consistently unchanged. This may be related to the type of cancer.

However, the Pt(IV)-EA conjugate containing a cDDP core and two axial ethacrynate ligands did not readily release a Pt(II) species to exert a synergistic cytotoxic effect and had poor aqueous solubility. To overcome these shortcomings, Lee et al. [94] redesigned a new Pt(IV) construct comprising a cDDP core with one axial ethacrynate ligand and one axial hydroxido ligand (Figure 14(**49**)). This compound was not only more soluble in the phosphate buffer at the required concentrations, due to the substitution of the hydrophobic ethacrynate ligand with the hydrophilic hydroxide moiety, but also more susceptible to reduction. Moreover, the novel complex not only remained the inhibitory activity of GST but also obviously reduced tumor growth in vivo in a chicken embryo with low toxicities compared with cisplatin, thus validating the new design strategy.

### 2.10. Targeting Glutamine (Gln) Receptor

**Figure 15:**
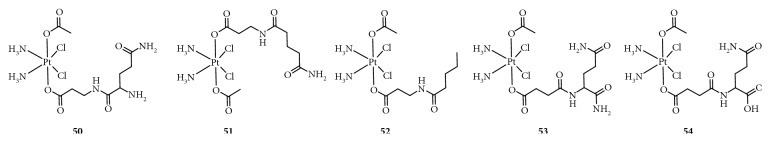
Structure of Pt(IV) prodrugs based on glutamine (Gln).

Glutamine (Gln), a nonessential amino acid, is abundant in the plasma, reaching 0.4–0.7 mM concentrations [95]. Gln plays an important role in the rapid growth of the tumor by providing nitrogen and carbon for the biosynthesis of components such as nucleotides and hexosamines [96]. Interestingly, in some cancer cells such as pancreatic, glioblastoma, leukemia, lung, and breast cancers, Gln deprivation induced cell apoptosis, which is known as “Gln addiction.” Based on this observation, targeting Gln addiction is an attractive strategy for cancer therapy. Ravera et al. [97] synthesized a small series of Gln- or Gln derivative-conjugated Pt(IV) complexes (Figure 15(**50**–**54**)). Unfortunately, these complexes were less potent than cisplatin, while showing decreased off-target accumulation. Therefore, new amino acid-conjugated Pt(IV) complexes need to be designed for targeting tumor cells.

### 2.11. Targeting Immunoregulation

**Figure 16:**
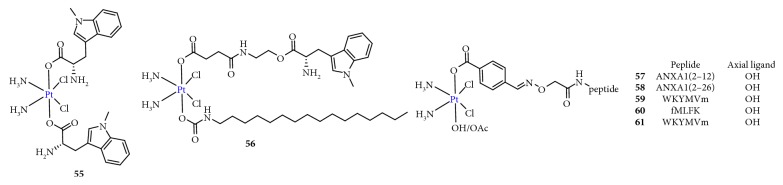
Structure of Pt(IV) prodrugs based on (D)-1-MT and formyl peptide receptor 1 and 2 (FPR1/2) peptide.

Because toxic drugs are considered immunosuppressive, the use of chemotherapy that targets the immune system was discounted [98]. In fact, clinical data have shown that platinum agents directly or indirectly act as immune effectors. Nonetheless, this fact has been ignored in the development of new therapeutics, and studies have primarily focused on targeting DNA. Presently, attractive immunotherapy approaches mainly include chimeric antigen receptor (CAR) T-cell therapies, cancer vaccines, dendritic cell therapies, and immune checkpoint inhibitors [99, 100]. The common inhibitory immune checkpoints include programmed cell death protein 1 (PD-1), cytotoxic T-lymphocyte-associated protein 4 (CTLA-4), T-cell immunoglobulin and mucin-domain containing-3 (TIM3), and indoleamine 2,3-dioxygenase (IDO) [101]. Targeted immune checkpoint therapy could increase the antitumor immune response by affecting T cells. Taking advantage of the potential synergy between platinum drugs and immune checkpoint inhibitors by developing combination therapies is likely to provide survival benefits to patients. Awuah et al. [102] adopted the Pt(IV) prodrug strategy to combine the immunomodulator (D)-1-MT (IDO inhibitor) with cisplatin to obtain the first immuno-chemotherapeutic agent for DNA cross-linking-induced apoptosis (Figure 16(**55** and **56**)). This complex effectively induced DNA damage, blocked IDO to prevent T-cell degradation, and downregulated the aryl hydrocarbon receptor (AHR) and IL-6, which are key genes involved in the autoregulation of constitutive IDO expression, resulting in T-cell proliferation. Wong et al. [103] synthesized an immuno-chemotherapeutic Pt(IV) prodrug using a formyl peptide receptor (FPR) 1/2 peptide combined with cisplatin (Figure 16(**57**–**61**)). The results showed that the FPR1/2 peptide not only efficiently delivered cisplatin to cancer cells, which overexpressed FPR1/2 on the surface, but also activated, as a potent immune adjuvant, an immune anticancer response. Therefore, combining chemotherapy with immunotherapy to achieve a therapeutic synergy is a feasible approach.

### 2.12. Multitargeting

**Figure 17:**
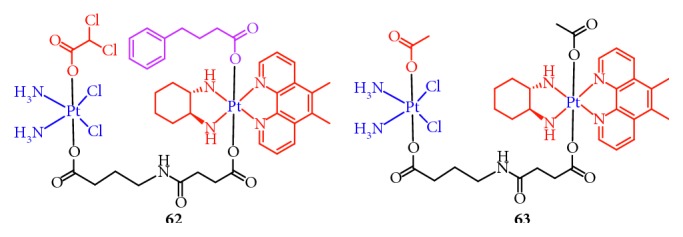
Structure of Pt(IV) prodrugs based on cisplatin, dichloroacetate (DCA), 4-phenylbutyric acid (PhB), and Pt56MeSS.

In general, “dual-action” Pt(IV) prodrugs are designed to treat cancers based on the combination of a single chemotherapeutic entity with a single bioactive entity. Recently, Petruzzella and colleagues [104] created a “quadruple-action” Pt(IV) prodrug that released four different bioactive moieties, cisplatin, DCA (PDK inhibitor), PhB (HDAC inhibitor), and Pt56MeSS, in KRAS-mutated cancer cells (Figure 17(**62** and **63**)). The data showed that this Pt(IV) prodrug significantly increased the cytotoxicity, 200–450-fold compared with that of cisplatin, in KRAS-mutated pancreatic and colon cancers, which resulted from synergism between DNA platination, HDAC inhibition, and action on the mitochondria. Compared to normal cells, the recognition capability of this Pt(IV) prodrug for cancer cells was 40-fold higher. The study provides a novel option for using Pt(IV)-based “quadruple-action” prodrugs as anticancer agents.

### 2.13. Targeting Cell-Surface Receptors

#### 2.13.1. Targeting Estrogen Receptor (ER) and Androgen Receptor (AR)

**Figure 18:**
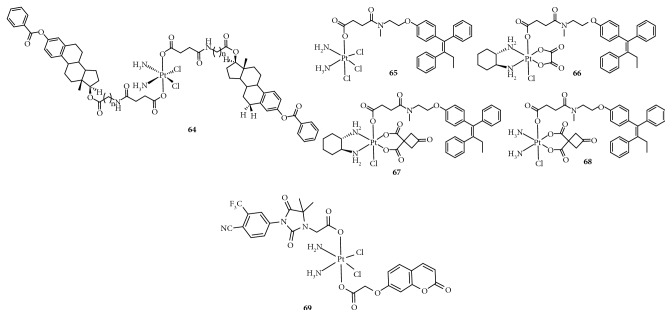
Structure of Pt(IV) prodrugs based on estrogen, tamoxifen, and androgen receptor (AR).

Barnes et al. [105] demonstrated that estrogen receptor- (ER-) positive cancer cells are sensitized to cisplatin by treatment with estrogen, which could shield cisplatin-DNA adducts from NER through overexpression of the high-mobility group box 1 (HMGB1) protein. HMGB1 is the most abundant and prominent member of the HMG family of ubiquitous and highly conserved nonhistone chromosomal proteins found in higher eukaryotes [106]. It has been reported that the level of chromosomal architectural protein HMGB1 is upregulated by estrogen and steroid hormones [107]. Importantly, HMGB1 binds specifically to the major cisplatin-DNA adducts, 1,2-intrastrand d(GpG) and d(ApG) cross-links, which comprise 90% of the lesions, inhibiting repair of DNA damage by the NER and sensitizing cancer cells to cisplatin [108]. Therefore, the authors designed a series of Pt(IV) derivatives conjugated to estrogen, called *bis*-estrogen-*cis*-diamminedichloroplatinum(IV) (BEP_n_, Figure 18(**64**)). The results showed that all BEP_n_ complexes induced overexpression of HMGB1 in ER^+^ MCF-7 cells compared with ER^−^ HCC-1937 cells, and BEP3 was nearly 2-fold more cytotoxic to ER^+^ MCF-7 cells than it was to ER^−^ HCC-1937 cells. These observations suggest that these complexes specifically to target ER^+^ cancer cells such as breast and ovarian cancer cells provide a direct strategy to enhance the antitumor effect of platinum complexes. In addition, Gou's group [109] reported conjugation of the Pt(IV) moiety to the ER modulator tamoxifen, named Tam-Pt(IV) (Figure 18(**65**–**68**)), which was selectively and efficiently taken up by ER^+^ positive tumor cells. In ER^+^ positive tumor cells, these Pt(IV) complexes could be activated by intracellular reducing molecules such as ascorbic acid and GSH to release the Pt(II) complex and tamoxifen moieties simultaneously and regain their pharmacological activity. Tamoxifen, as a first-line endocrine therapy, has been widely used for patients with all stages of ER^+^ breast cancer; however, its use has been limited by intrinsic and acquired drug resistance. However, these Tam-Pt(IV) compounds were found to reverse the tamoxifen resistance of breast cancer, particularly that of MCF-7 (ER*α*^+^ breast cancer cells), but it was less effective toward MDA-MB-231 (ER*α* negative breast cancer cells). Therefore, the combination of estrogen modulators and the platinum moiety could enhance selective development of platinum in ER^+^ tumors and possibly expand the clinical scope of the ER ligand in resistant breast tumors.

Currently, PC is one of the leading causes of cancer-related deaths in men worldwide, especially castration-resistant PC (CRPC), with poor prognosis, metastatic spreading, and a median survival range from 18 to 24 months. Although the mechanisms responsible for the progression of PC to CRPC are not well known, the expression level of the AR is reported to be approximately 6-fold higher in CRPC than hormone-sensitive PC, suggesting that AR protein is essential for CRPC to adapt to the low levels of androgens in PC. Therefore, Qin and colleagues [110] reported a Pt(IV)-based prodrug targeting AR (Figure 18(**69**)). This compound was found to display satisfactory AR binding affinity and selectively accumulated in LNCaP (AR+) cells, other than PC-3 (AR−) cells, which could be effectively visualized. Moreover, it exhibited excellent anticancer activity superior to that of cisplatin.

#### 2.13.2. Targeting Membrane-Bound Heat Shock Protein 70 (memHSP70)

Heat shock protein 70 (HSP70), a stress-inducible chaperone, is overexpressed in many cancers including colorectal cancer. Clinical data has shown that 50% of patient tumors were characterized by obvious overexpression of a membrane-bound form of HSP70 (memHSP70) [111]. Normal cells maintain memHSP70 homeostasis, while in tumor cells, the expression of memHSP70 is upregulated, which is associated with cancer progression and poor prognosis. Gehrmann et al. [111] reported that TPP, an HSP70-derived 14-mer peptide, could rapidly bind to an epitope in the oligomerization domain of HSP70 and be taken up by internalization, suggesting that TPP may have the potential to target cancer cells that express HSP70. Based on this hypothesis, McKeon et al. [112] combined oxaliplatin with TPP to synthesize a novel Pt(IV) prodrug. This complex targeted cancer cells through specific binding and internalization by memHSP70^+^ tumor cells.

#### 2.13.3. Targeting Glucose Transporters

**Figure 19:**
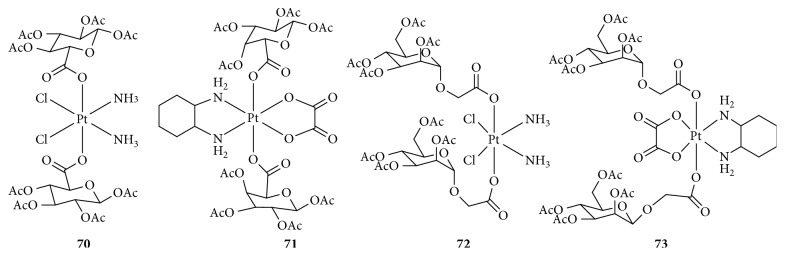
Structure of glycosylated Pt(IV) prodrugs.

Carbohydrates play important roles in various biological processes and can significantly promote the unrestricted proliferation of cancer cells. Carbohydrate uptake mainly involves two types of proteins: glucose transporters (GLUTs) and sodium-dependent glucose transporters (SGLTs), which are overexpressed in tumor cells because of the Warburg effect and are prognostic indicators for cancer, especially GLUT1 [113, 114]. Therefore, the combination of a sugar and antitumor agents has become an attractive strategy for cancer therapy. It has been reported that novel glycosylated Pt(II) agents showed obviously reduced toxicity and enhanced antitumor activity. Recently, several glycosylated Pt(IV) prodrugs have been synthesized and explored for their bioactivity [115–117]. These glycosylated Pt(IV) complexes (Figure 19(**70**–**73**)), with various sugars (glucose, mannose, galactose, or rhamnose) conjugated to a platinum core (cisplatin or oxaliplatin), were found to be comparable or even superior to cisplatin or oxaliplatin. The IC_50_ and cytoplasmic concentrations of glycosylated Pt(IV) complexes varied depending on the axial ligand, Pt core, or cell phenotype. In general, Pt(IV) complexes with the cisplatin core were more effective than their oxaliplatin counterparts, and mannose, galactose, or rhamnose were more potent than glucose. Moreover, Ma et al. [116] evaluated the effects of glycosylated Pt(IV) complexes on GLUT1. The results showed that these complexes were GLUT and organic cation transporter (OCT) substrates and contributed greatly to targeted therapy, although it had been previously reported that Pt(IV) complexes protected by acetyl groups did not have such properties. The precise mechanism needs to be further studied.

#### 2.13.4. Targeting Chlorotoxin (CTX) Receptors

**Figure 20:**
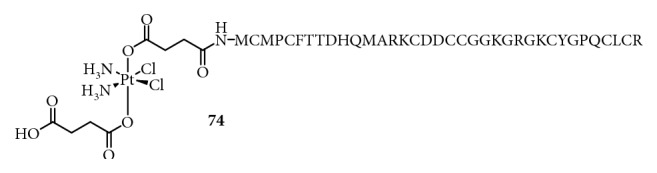
Structure of Pt(IV) prodrugs based on chlorotoxin (CTX).

Chlorotoxin (CTX, TM601) [118], a 36-amino-acid peptide with four disulfide bridges, was extracted from the venom of the giant yellow Israeli scorpion *Leiurus quinquestriatus hebraeus*. It has been reported that CTX binds to matrix metalloproteinase 2 (MMP2) or chloride ion channels. In general, the level of MMP2 increases, and chloride ion channels are overexpressed in cancers [119]. In addition, the annexin A2 protein has also been reported as a receptor for CTX [120]. These proteins are attractive targets for the development of targeted drugs. Graf et al. [121] have used CTX as a carrier for cisplatin and synthesized Pt(IV)-CTX conjugates for targeting cancer cells (Figure 20(**74**)). The results showed an increased efficacy of the platinum complex in three different cell lines compared to that of CTX or Pt(IV); however, the efficacy was lower than that of cisplatin.

#### 2.13.5. Targeting Integrins

**Figure 21:**
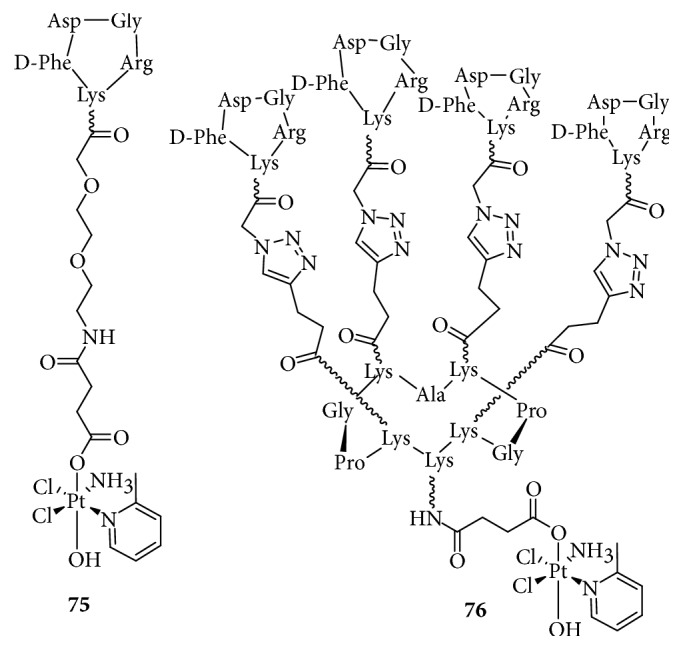
Structure of Pt-c(RGDfk) and Pt-RAFT-{c(RGDfk)}4.

Among the receptors overexpressed on tumor cells, integrins are attractive pharmacological targets. They are transmembrane cell adhesion glycoproteins involved in the migration, invasion, proliferation, and survival of tumor cells [122]. In particular, *α*_V_*β*_3_ and *α*_V_*β*_5_ integrins are frequently overexpressed in tumor endothelial cells in the lung, breast, melanoma, prostate, ovarian carcinoma, and brain tumors. Studies have shown that RGD is efficiently recognized by *α*_V_*β*_3_ and *α*_V_*β*_5_ integrins [123]. Therefore, using the high affinity of RGD to *α*_V_*β*_3_ and *α*_V_*β*_5_ integrins, researchers [124–127] synthesized some Pt(IV) prodrugs based on RGD-containing peptides or peptidomimetics to deliver platinum drugs to cancer cells or for tumor imaging (Figure 21(**75** and **76**)). These complexes effectively targeted and massively accumulated in cancer cells to induce tumor cell death with low toxicity to normal cells. In these complexes, RGD mainly serves as a carrier for drug delivery.

## 3. Conclusions

Although cisplatin is widely used in cancer treatment, its drawbacks such as acquired resistance limits its clinical applications and has led to the development of novel drugs with improved properties. To overcome the shortcomings of cisplatin, one approach is the development of Pt(IV) prodrugs with two axial ligands, which exhibit inertness before entering cells and minimize unwanted interactions with nucleophiles in the serum, thus reducing side effects and enhancing bioavailability. In recent years, multiple Pt(IV) prodrugs have been synthesized through modification of the two axial ligands to improve or confer particular features such as hydrophobicity, targeted delivery, photosensitivity, reduction quality, and dual or multiple functions. This review summarized the recent progress in the development of Pt(IV) prodrugs conjugated with biologically active ligands. Although such prodrugs were synthesized to exert dual function, these complexes frequently displayed multiple functions. Thus, Pt(IV) complexes conjugated with HDAC inhibitors not only affect DNA and HDAC activities but also influence related apoptosis pathways such as the mitochondrial pathway. The regulation of intracellular signals involves a vast network, but Pt(IV) prodrugs conjugated with biologically active ligands could, to some extent, increase accumulation in target tissue and antitumor activity, reduce toxicity, and overcome drug resistance. Therefore, Pt(IV) prodrugs conjugated with bioactive ligands are still considered a promising strategy for cancer therapy.
